# Hemophagocytic inflammatory syndrome in ADA-SCID: report of two cases and literature review

**DOI:** 10.3389/fimmu.2023.1187959

**Published:** 2023-06-26

**Authors:** Elena Sophia Fratini, Maddalena Migliavacca, Federica Barzaghi, Claudia Fossati, Stefania Giannelli, Ilaria Monti, Miriam Casiraghi, Francesca Ferrua, Salvatore Recupero, Giulia Consiglieri, Valeria Calbi, Francesca Tucci, Vera Gallo, Maria Ester Bernardo, Sabina Cenciarelli, Monica Palmoni, Margherita Moni, Luca Galimberti, Marzia Duse, Lucia Leonardi, Elena Sieni, Elena Soncini, Fulvio Porta, Lucia Dora Notarangelo, Raffaella De Santis, Saverio Ladogana, Alessandro Aiuti, Maria Pia Cicalese

**Affiliations:** ^1^ Pediatric Immunohematology and Bone Marrow Transplantation Unit, IRCCS San Raffaele Scientific Institute, Milan, Italy; ^2^ Università Vita-Salute San Raffaele, Milan, Italy; ^3^ San Raffaele Telethon Institute for Gene Therapy (SR-Tiget), IRCCS San Raffaele Scientific Institute, Milan, Italy; ^4^ Department of Pediatrics, La Sapienza University of Rome, Rome, Italy; ^5^ Paediatric Haematology/Oncology Department, Meyer Children’s University Hospital, Florence, Italy; ^6^ Pediatric Oncology-Haematology and Bone Marrow Transplantation (BMT) Unit, Spedali Civili di Brescia, Brescia, Italy; ^7^ Medical Direction, Children’s Hospital, ASST-Spedali Civili, Brescia, Italy; ^8^ Paediatric Onco-Haematology Unit, “Casa Sollievo della Sofferenza” Hospital, IRCCS, San Giovanni Rotondo, Italy

**Keywords:** hemophagocytic inflammatory syndrome, hemophagocytic lymphohistiocytosis (HLH), SCID, ADA-SCID, primary immunodeficiency

## Abstract

Hemophagocytic inflammatory syndrome (HIS) is a rare form of secondary hemophagocytic lymphohistiocytosis caused by an impaired equilibrium between natural killer and cytotoxic T-cell activity, evolving in hypercytokinemia and multiorgan failure. In the context of inborn errors of immunity, HIS occurrence has been reported in severe combined immunodeficiency (SCID) patients, including two cases of adenosine deaminase deficient-SCID (ADA-SCID). Here we describe two additional pediatric cases of ADA-SCID patients who developed HIS. In the first case, HIS was triggered by infectious complications while the patient was on enzyme replacement therapy; the patient was treated with high-dose corticosteroids and intravenous immunoglobulins with HIS remission. However, the patient required HLA-identical sibling donor hematopoietic stem cell transplantation (HSCT) for a definitive cure of ADA-SCID, without HIS relapse up to 13 years after HSCT. The second patient presented HIS 2 years after hematopoietic stem cell gene therapy (GT), secondarily to Varicella-Zoster vaccination and despite ^CD4+^ and ^CD8+^ lymphocytes’ reconstitution in line with other ADA SCID patients treated with GT. The child responded to trilinear immunosuppressive therapy (corticosteroids, Cyclosporine A, Anakinra). We observed the persistence of gene-corrected cells up to 5 years post-GT, without HIS relapse. These new cases of children with HIS, together with those reported in the literature, support the hypothesis that a major dysregulation in the immune system can occur in ADA-SCID patients. Our cases show that early identification of the disease is imperative and that a variable degree of immunosuppression could be an effective treatment while allogeneic HSCT is required only in cases of refractoriness. A deeper knowledge of immunologic patterns contributing to HIS pathogenesis in ADA-SCID patients is desirable, to identify new targeted treatments and ensure patients’ long-term recovery.

## Introduction

Hemophagocytic lymphohistiocytosis (HLH) is a major dysregulation of the immune system. Consequent hypercytokinemia and auto-inflammatory syndrome can evolve, if untreated, into multiorgan failure and death. Diagnosis usually comes delayed, with an impact on patient survival and long-term complications, so early recognition of the disease is crucial. For this purpose, the Histiocyte Society established new diagnostic guidelines in 2004, where three additional criteria were introduced; low/absent NK cell activity, hyperferritinemia, and high-soluble interleukin-2-receptor levels (IL2r) (1). Current treatment regimens for HLH include immune suppressants, immunomodulators, and definitive cure by allogeneic hematopoietic stem cell transplantation (HSCT) in selected patients.

Familial HLH (FHL) usually affects infants that carry a spectrum of inherited defects in cytotoxic lymphocyte function, often triggered by underlying uncontrolled infections. HLH may also arise as a secondary condition resulting from persistent antigen stimulation due to autoimmune disease or malignancy (2). More widely, any defect in T cells or NK/phagocytic function or major constitutional immunological abnormality may contribute to HLH pathogenesis. In this view, studying HLH has contributed to shedding light on the immune system functioning and nowadays gives new leads to advances in the understanding of the pattern of perpetuation of the inflammation, uncovering possible targets in the pathway for autoinflammatory diseases.

A secondary form of HLH, Hemophagocytic inflammatory syndrome (HIS), has been described in selected Inborn Errors of Immunity (IEI) as in defects of *MAGT1*, *GATA2 WAS*, and *ADA2* genes, but also in patients with chronic granulomatous disease (CGD) and severe combined immunodeficiencies (SCID) (3, 4), as a deficit of *ILRG2, RAG1, IL7RA, CD3E*, and Adenosine Deaminase-SCID (ADA-SCID) (5, 6).

ADA‐SCID is a life‐threatening immunodeficiency, characterized by lymphopenia in B/T/NK subpopulations, failure to thrive, severe infections, and autoimmunity (7). Allo-HSCT and Strimvelis an *ex-vivo* retroviral hematopoietic stem cell gene therapy (HSC-GT) approved in the EU, are standard-of-care treatments for ADA-SCID (8, 9).

Patients with HIS in the context of IEIs present major diagnostic and therapeutic implications, related to the branch of the immune system involved in the HLH genesis and to the treatment choice, spanning from immunosuppressive agents, with related increased infection risk, to allo-HSCT in refractory cases.

In our study, we described two HIS cases emerging in ADA-SCID patients. Moreover, we provide a report of two cases described in the literature.

## Results

We evaluated retrospectively HLH-2004 criteria in two ADA-SCID children followed in San Raffaele Hospital between 2005 and 2020. Both patients had a previous history of enzyme replacement therapy (ERT) with Polyethylene glycol-modified adenosine deaminase (PEG-ADA) before HIS onset and survived without recurrence. Therapeutic strategies were discussed with the families and treatments did not lead to adverse or unanticipated events. Family adherence to physicians’ indications was complete. No persistent organ damage or cognitive/mental health issues related to the hospitalization or to the medications were documented.

**Case 1.** A 2-year-old Italian child affected by ADA-SCID was referred to our center to undergo experimental HSC-GT (10). The patient was on oral steroids for chronic autoimmune hemolytic anemia (AHA). A central venous line catheter was inserted under sedation and the procedure was complicated by right pneumothorax, promptly drained. Five days after surgery the child presented with fever and a diffuse maculopapular rash on the trunk, eyelids, hands, and feet. Contextually, high inflammatory indexes were observed (CRP>500 mg/L, nv <0.5), with mild hepatomegaly confirmed by ultrasound. The fever continued unremittingly in the following days. The blood cultures were positive for *Staphylococcus epidermidis and hominis*, therefore intravenous antibiotic therapy (vancomycin, rifampin, and imipenem), and empirical intravenous immunoglobulins (IVIg) and antifungal treatment were started. Heart US showed possible vegetation on the central venous line tip in the right atrium, therefore, suspected infectious endocarditis, Port-a-cath was removed. After surgery, left pleural hematic effusion was evident, with consumptive coagulopathy (INR 2.1, nv 0.8-1-2, D dimer 13 µg/mL nv 0.27-0.77, mild thrombocytopenia). Three weeks after surgery, the patient’s clinical conditions worsened, with spiking high fever and dyspnea. A total body CT scan was performed documenting recrudescence of left pleural effusion with subsequent drainage of bloody fluid. The child, upon family request, was transferred to the referring hospital. Despite an improvement in general condition, the patient continued to have fever, diffuse skin rash, and hepatomegaly. Laboratory exams (**Table 1**) further showed increased neutrophil counts, lymphopenia, hypertriglyceridemia and hyperfibrinogemia, D Dimer 114.9 (nv <0.5), and CRP 160 mg/l (nv <0.5). HIS was empirically diagnosed despite HLH criteria not being fully evaluated due to the urgent need for patient’s management, therefore steroid therapy with Methylprednisolone 2 mg/kg/day was started, further increased up to 5 mg/kg/day with prompt remission of symptoms, despite the persistence of systemic signs of inflammation. The prominent hypercoagulative state was complicated by endocaval thrombi along the course of the femoral central venous line, requiring antithrombotic therapy with good response.

**Table 1:** Laboratory evidence and clinical features of HIS in ADA-SCID patients.

		Case 1	Case 2	Case 3 (5)	Case 4 (6)
Laboratory criteria	**Cytopenia**	**Yes Bilinear**	**Yes Bilinear**	**Yes Trilinear**	**Yes Bilinear**
	**Tryglicerids mg/dl (n.v <150)**	**496**	**549**	**383**	**532**
	**Fibrinogen mg/dl (n.v 150-400)**	684	**123 (nadir)**	754	333
	**Hemophagocitosis in the bone marrow**	**Reticular cells in active phagocytosis of cell debris**	Not present	**Active hemophagocytosis**	Not performed
	**Ferritin ng/dl (n.v. 30-400)**	Not performed	**25547**	**18000**	13797
	**IL-2R pg/ml (n.v 600-2000)**	Not performed	**49934**	**16809**	Not performed
	**Natural killer activity**	Not performed	Not evaluable	Normal	Not performed
	**Perforin analysis**	Not performed	Adequate	Normal	Not performed
Clinical criteria and additional characteristics	**Fever**	**Yes**	**Yes**	**Yes**	**Yes**
	**Splenomegaly**	No	No	**Yes**	**Yes**
	**Hepatomegaly**	Yes	Yes	Yes	Yes
	**Skin rash**	Yes	Yes	Yes	No
	**Arthritis**	No	Yes	No	No
	**Disseminated intravascular coagulation**	Yes	No	Yes	Yes
	**Acute kidney injury**	No	No	No	Yes

Text in bold highlights HLH-2004 satisfying criteria$.

$ HLH-2004 diagnostic criteria_3_: ≥5 of 8 criteria are diagnostic: fever, splenomegaly, bilinear or trilinear cytopenia (hemoglobin <90 g/L, platelets <100 x 10 (9)/L, neutrophils <1.0 x 10 (9)/L), hypertriglyceridemia and/or hypofibrinogenemia, hemophagocytosis on bone marrow, low/absent NK-cell activity, hyperferritinemia, high soluble interleukin-2 receptor levels.

ERT was stopped for two months and IVIg was administered once a week for one month. After two months of high-dose corticosteroid therapy, progressive tapering was started, with suspension 5 months after HIS onset, contextual complete resolution of the clinical signs, and laboratory tests normalization. After complete resolution of the disease (9 months after the onset), the patient was treated with HSC-GT in our center receiving 10.7 * 10^6 CD34+^/kg with a vector copy number (VCN) equal to 0.12 cp/cell (11). Unfortunately, graft failure of gene-corrected cells occurred (10), thus the child reintroduced ERT and subsequently received an HLA identical sibling HSCT (unavailable at the time of GT) without conditioning at the referring Centre. HSCT resulted in successful engraftment without recurrence of HIS, and 13 years after HSCT.

**Case 2.** A 4-year-old male child affected by ADA-SCID was started on PEG-ADA at 3 months of life and treated in 2017 with Strimvelis (12) at 1 year of age. Medicinal Product dose and VCN were in line with the subjects treated in the clinical development program (13).

**Figure 1:**
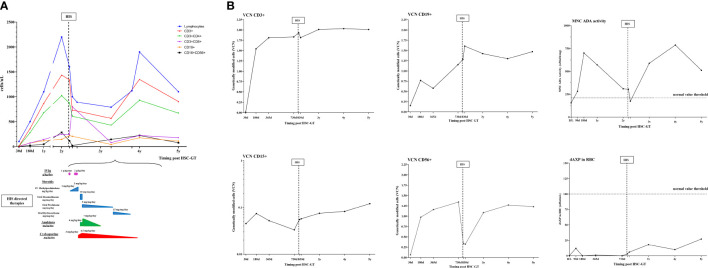
Schematic representation of immunological, molecular, and biochemical parameters and HIS-directed therapy in case 2. **(A)** Kinetics of lymphocytes subpopulations (^CD3+,CD3+CD4+,CD3+CD8+,CD19+,CD16+CD156+^) post HSC-GT over time (upper panel) and HIS-directed therapies (numbers indicate starting dose and maximum dose used) (lower panel); **(B)** Vector copy number in lymphocytes subpopulations (^CD3+,CD19+,CD56+^) and granulocytes (^CD15+^) and ADA-SCID biochemical analysis (ADA activity on mononuclear cells and purine metabolites in red blood cells). MNC, mononucleated cells; dAXP, Deoxyadenosine nucleotide percentage; RBC, red blood cells; d, days; B/L, baseline.

Clinical history following infusion of Strimvelis was unremarkable, without autoinflammatory/autoimmune issues, until 2 years of follow-up. The patient’s lymphocyte counts and subpopulations were progressively improving, although mild lymphopenia for their age was still present (**Figure 1**).

Molecular exams showed transduced cells in all hematopoietic lineages, good enzyme activity, and satisfactory detoxification. ^CD3+CD4+^ and ^CD16+CD56+^ were within normal limits, with a good naive/memory ratio. ^CD3+CD8+^ and B cells were lower than normal for age in particular ^CD3+^ count was more than 0.5 × 10^9^/l, and ^CD3+CD8+^ count was more than 0.2 × 10^9^/l with adequate proliferative response to mitogens. (**Figure 1**). Ig supplementation was stopped 8 months post-GT and the child received inactivated vaccines (hexavalent, pneumococcus, and meningococcus ACWY and B) resulting in a robust antibody response.

As part of the local standard vaccination schedule, anti-Varicella zoster (VZV) immunization was performed 2 years after GT. One week after vaccination the child presented a low-grade intermittent fever lasting two weeks. Because of this fever, left ankle monoarticular arthritis, and a diffuse salmon maculopapular rash, the child was admitted to a local hospital.

Blood exams revealed hyperferritinemia (**Table 1**), therefore Methylprednisolone 1 mg/kg/day was started, along with intravenous broad-spectrum antibiotics and antiviral therapy.

Prompt amelioration of the child’s conditions was observed, however, after a week the fever pattern worsened, with a persistent rise in inflammatory indexes. Increased doses of Methylprednisolone at 2mg/kg/day and IVIg at 1g/kg were administered. Bone marrow biopsy did not show signs of hemophagocytosis.

The child was transferred for additional immunologic investigations to our unit and he was started on oral dexamethasone 10 mg/m2/d. At admission, laboratory exams, reported in **Table 1**, showed: elevated inflammatory indexes with CRP 25 mg/L (nv <0.5), ESR 57 mm/1h (nv 1-15), mild anemia, and thrombocytopenia. On immunophenotype analysis reduction in lymphocyte counts, in particular of ^CD56+^ was observed, with a decreased VCN in ^CD56+^ subpopulation (**Figure 1**). VZV DNA on plasma was negative (**Table 1**). Coagulopathy was observed with APTT < 23 sec, and factor VIII (300%, nv 65-140) without evidence of thrombosis. In the following months, after the progressive tapering of corticosteroid therapy, relapse of fever and a diffuse maculopapular rash was observed. Heart US showed normal coronary arteries echostructure. Diagnosis of HIS triggered by Varicella zoster vaccination was formulated, fulfilling 5 out of 8 HLH-2004 criteria. Immunosuppressive therapy was optimized by increasing the dose of corticosteroids, starting Cyclosporin A (CyA) and an anti-IL1 receptor antagonist (Anakinra) (**Figure 1**).

Improvement of clinical conditions and normalization of inflammatory and liver function indexes were observed in the following months. Anakinra and CyA were stopped after 12 and 18 months from HIS onset, respectively. At the latest follow-up, following discontinuation of immunosuppression, lymphocytes were below normal for age, within the lower range of ADA-SCID patients treated with HSC-GT, with normal response to mitogens. Furthermore, persistence of gene-corrected cells and recovery of transduced ^CD56+^ cells was observed.

Details of **case 3** and **case 4**, identified in the literature, are provided in **Tables 1** and **2**.

**Table 2:** Patients’ characteristics.

Age of HIS	Case 1	Case 2	Case 3 (5)	Case 4 (6)
	2 years	4 years 1 month	3 years 11 months	3 months
Previous treatment for ADA-SCID	ERT	ERT HSC-GT (Strimvelis)	ERT HSC-GT (Strimvelis) Haploidentical αβ+ T-cell and ^CD19+^ B-cell depleted (father)	None
Concomitant infection/Vaccination	*Staphylococcus epidermidis and hominis*	Live attenuated Varicella zoster	Adenovirus *Calmette-Guérin Bacillus* Aspergillus spp	Cytomegalovirus
HLH-2004 criteria	3/8	5/8	5/8	5/8
HIS directed therapy	Steroid IVIg ERT stop for 2 months	Steroids Anti IL-2 receptor antagonist (Anakinra) Cyclosporine A IVIg	Steroids Haploidentical HSCT (from mother-Etoposide based conditioning) Emapalumab	None
Age at ADASCID diagnosis	5 months of life	3 months of life	1 year and 1 month	3 months
Treatments after HIS for ADASCID	ERT HSC-GT Allogenic HSCT	None	Haploidentical αβ+ T-cell and ^CD19+^ B-cell depleted (maternal)	None
Outcome	HIS remission (18 years from onset) Alive with full chimerism (follow up 13 years post HSCT)	HIS remission (follow up of 3 years) Alive without ERT re start (6 years from HSC-GT)	HIS remission (4 years from onset) Alive without ERT re-start (4 years from allo-HSCT)	Death

## Discussion

In ADA-SCID, HIS occurred as a complication of various immunologic triggers such as vaccination, infections, and post-HSCT, as in **case 3** (6), but also as a first finding of IEI with a still unknown diagnosis, as in **case 4** (5). Misdiagnosis with a septic status complicated the children’s treatments. In fact, the diagnosis was delayed in all cases because immunologic dysfunction was suspected only when the patients’ clinical conditions deteriorated.

The characteristics of our two patients were in line with the other two cases reported in the literature (**Cases 3 and 4**) (**Table 2**). In all cases, increased liver function enzymes, LDH, ferritinemia, and trygliceridemia with a procoagulant state were observed. IL2r, when tested, was dramatically elevated in all the patients (**Table 1**). Unremittent fever, skin rash, and hepatomegaly were common to all children, while additional symptoms were arthritis, disseminated intravascular coagulation, and acute kidney failure. Cytopenia (Bi/Trilinear) was a constant finding, whether associated with clear hemophagocytosis signs or not, as observed in **case 3**. Pancytopenia was present in **case 3**, while mild anemia and thrombocytopenia were present in **cases 1 and 4**.

Some of the most updated tests specific to HLH were performed in **cases 2** and **3** (i.e. perforin analysis, while NK activity was not feasible due to lymphopenia in **case 2**). The patient in **case 1** did not undergo testing because of the lack of published HLH guidelines at that time (1). Genetic analysis for familiar HLH was performed and found negative in **case 4**.

Autoimmunity is known to be associated with ADA deficiency (14) and **case 1** displayed overt autoimmune manifestations before the onset of HIS (AHA), while **case 2** presented a positive family history (father with intestinal bowel disease diagnosis).

In our cases, different therapeutic approaches were successful in managing HIS, and an escalation strategy to more aggressive approaches was chosen, to guarantee safety. Eventually, haploidentical HSCT was performed as a rescue strategy in **case 3** (6).

In **case 1** high-dose corticosteroids, IVIg, and a short washout from ERT were attempted and were sufficient to silence HIS. Low-dose Busulfan conditioning performed prior to HSC-GT could have had a role in maintaining remission. However, due to GT failure, the patient received a subsequent HLA-identical HSCT without conditioning after 2 years from HSC-GT, with successful outcome and with no HIS recurrence.

Post HSC-GT, rescue HSCT was performed in **case 3** due to a refractory HIS. Haplo-HSCT was primed with emapalumab and conditioning with etoposide, cyclophosphamide, anti-thyroglobulin, and CyA, due to multiple episodes of graft rejection (7). The patient is currently 4 years post-HSCT in good clinical conditions, without HIS relapse or cGvHD.

Interestingly, the cause of failure of HSC-GT engraftment in **cases 1 and 3** could have been multifactorial; ongoing infections as well as the immunodysregulated background of both patients could have negatively influenced the quality of the HSC and BM microenvironment, possibly contributing to HIS pathogenesis.

Our data in **case 2** provide also useful insights into the kinetics of gene-corrected cells during the occurrence of HIS. Despite good immunological reconstitution, HIS was accompanied by a reduction in NK absolute count in the context of generalized lymphopenia, with a relative increase of untransduced NK gene-corrected cells (lower VCN) (**Figure 1**). We speculate that mostly residual uncorrected NK cells could have contributed to sustaining HIS priming and maintenance, in the presence of a powerful immunologic trigger such as the VZV immunization. In this case, HIS was controlled by immunosuppressive drugs (i.e. corticosteroids, Anakinra, CyA), suggesting that despite the initial immune dysregulation, gene-corrected cells were sufficient to restore normal immune functions.

The occurrence of HIS manifestations in ADA-SCID confirms that these patients carry a significant burden of immune dysregulation. Autoimmunity/autoinflammation manifested in literature both in patients on ERT and in those who have undergone BMT or GT, having achieved variable degrees of immune reconstitution (14).

It remains unclear which mechanisms engage in HIS development, but it is highly reasonable that both T lymphocytes and NK cells could play a role, failing to control excessive inflammatory responses and leading to a cytokine storm.

In conclusion, our series indicates that the occurrence of unexplained fever with marked hyperferritinemia, sudden cytopenias, or coagulopathy in a patient with ADA-SCID should be urgently investigated for HIS. HIS may be more common in ADA-SCID and IEIs than recognized, as clinical and laboratory features in these patients may camouflage behind the occurrence of severe infectious complications associated with the underlying genetic condition.

## Conclusion

We report two rare cases of HIS associated with ADA-SCID and review the existing literature. Both HIS diagnosis and treatment resulted challenging in ADA-SCID patients. Characterization of the multifactorial innate and adaptive cellular immunity dysregulation processes will be important to better understand the pathogenetic mechanism of HIS in ADA-SCID and other IEIs. HIS treatment remains challenging, spanning from immunosuppressive/immunomodulatory drugs to a complete immune-hematological system replacement by allo-HSCT, which becomes imperative in case of poor response or refractoriness. Ultimately, once again congenital immunodeficiencies show to have an unbreakable bond with HIS.

## Data availability statement

The original contributions presented in the study are included in the article/supplementary material. Further inquiries can be directed to the corresponding author.

## Ethics statement

Ethical review and approval was not required for the study on human participants in accordance with the local legislation and institutional requirements. Written informed consent to participate in this study was provided by the participants’ legal guardian/next of kin. Written informed consent was obtained from the participant/patient(s) for the publication of this case report.

## Author contributions

ESF and MMi provided clinical care for the patient, participated to data collection and analysis and wrote the manuscript. BF,MC, FF, RS, GC, VC, FT, VG, MB, SC, LL, MD provided clinical care for the patient. ESo, LN, FP, DR, SL provided clinical care for the patient and participated to data collection. MP, MMo, LG contributed to manuscript drafting and contributed to data collection. FC contributed to data collection. SG, IM and ESi performed molecular and immunological studies on patient’s samples. AA is the ADASCID gene therapy clinical trial principal investigator, contributed to data interpretation and manuscript writing. MC is the ADASCID gene therapy clinical trial investigator, coordinated data collection, analysis and interpretation, provided clinical care for the patient, and wrote the manuscript. All authors contributed to the article and approved the submitted version.
